# Preoperative detection of TERT promoter and BRAF^V600E^ mutations in papillary thyroid carcinoma in high-risk thyroid nodules

**DOI:** 10.20945/2359-3997000000116

**Published:** 2019-03-18

**Authors:** Tatiana Marina Vieira Giorgenon, Fabiane Tavares Carrijo, Maurício Alamos Arruda, Taíse Lima Oliveira Cerqueira, Haiara Ramos Barreto, Juliana Brandão Cabral, Thiago Magalhães da Silva, Patrícia Künzle Ribeiro Magalhães, Léa Maria Zanini Maciel, Helton Estrela Ramos

**Affiliations:** 1 Universidade de São Paulo Universidade de São Paulo Faculdade de Medicina de Ribeirão Preto Departamento de Medicina Interna Ribeirão Preto SP Brasil Departamento de Medicina Interna, Faculdade de Medicina de Ribeirão Preto da Universidade de São Paulo (FMRP-USP), Ribeirão Preto, SP, Brasil; 2 Universidade Federal da Bahia Universidade Federal da Bahia Instituto de Ciências da Saúde Laboratório de Estudo da Tireoide Salvador BA Brasil Departamento de Biorregulação, Laboratório de Estudo da Tireoide, Instituto de Ciências da Saúde, Universidade Federal da Bahia (UFBA), Salvador, BA, Brasil; 3 Universidade Federal da Bahia Universidade Federal da Bahia Instituto de Ciências da Saúde Programa de Pós-graduação em Processos Interativos dos Órgãos e Sistemas Salvador BA Brasil Programa de Pós-graduação em Processos Interativos dos Órgãos e Sistemas, Instituto de Ciências da Saúde, Universidade Federal da Bahia (UFBA), Salvador, BA, Brasil; 4 Liga Bahiana Contra o Câncer Hospital Aristides Maltez Departamento de Patologia Salvador BA Brasil Departamento de Patologia, Hospital Aristides Maltez, Liga Bahiana Contra o Câncer, Salvador, BA, Brasil; 5 Universidade Estadual do Sudoeste da Bahia Universidade Estadual do Sudoeste da Bahia Departamento de Ciências Biológicas Jequié BA Brasil Departamento de Ciências Biológicas, Universidade Estadual do Sudoeste da Bahia (UESB), Jequié, BA, Brasil

**Keywords:** TERT, *BRAF* ^V600E^, papillary thyroid carcinoma

## Abstract

**Objectives:**

This observational study analyzed telomerase reverse transcriptase (*pTERT*) mutations in 45 fine-needle aspiration (FNA) specimens obtained from thyroid nodules followed by postoperatively confirmation of papillary thyroid cancer (PTC) diagnosis, examining their relationship with clinicopathologic aspects and the *BRAF*^V600E^ mutation.

**Subjects and methods:**

Clinical information was collected from patients who presented to Ribeirao Preto University Hospital for surgical consultation regarding a thyroid nodule and who underwent molecular testing between January 2010 to October 2012. Tests included a DNA-based somatic detection of *BRAF*^V600E^ and *pTERT* mutations.

**Results:**

We found coexistence of *pTERT*^C228T^ and *BRAF*^V600E^ mutations in 8.9% (4/45) of thyroid nodules. All nodules positive for *pTERT* mutations were *BRAF*^V600E^ positives. There was a significant association between *pTERT*^C228T^/*BRAF*^V600E^ with older age and advanced stage compared with the group negative for either mutation.

**Conclusions:**

This series provides evidence that FNA is a reliable method for preoperative diagnosis of high-risk thyroid nodules. *pTERT*^C228T^/*BRAF*^V600E^ mutations could be a marker of poor prognosis. Its use as a personalized molecular medicine tool to individualize treatment decisions and follow-up design needs to be further studied.

## INTRODUCTION

Papillary thyroid cancer (PTC) risk stratification and prognostication has been normally placed on clinicopathologic aspects, which are usually unreliable and presurgically nonexistent (1). In recent years, molecularly established prognostication for PTC has been broadly advised (2–4). The role of *BRAF*^V600E^-mutation test in bettering the preoperative premonition of thyroid nodules US guided fine-needle aspiration (FNA) is dubious in terms of the prognostic accuracy of *BRAF*^V600E^ mutations in PTC (5,6).

Telomerase reverse transcriptase (*TERT*) has been known to play a decisive role in cellular immortality by preserving the telomere length at the end of chromosomes and in encouraging other cellular functions such as proliferation and cell cycles (7). *TERT* gene promoter mutations (*pTERT*) increment the transcriptional activities of the *TERT* and have been connected to malignant tumors with superlative recurrence and lower survival in PTC (7,8). Only three studies preoperatively investigated *pTERT* mutations in PTC patients and proposed that the awareness of the mutation status might guide the amplitude of initial surgery (9–11). Coexistence of *BRAF*^V600E^ and *pTERT* mutations leads to a more aggressive subgroup of PTC, whereas the two mutations alone have relatively less impact on the aggressiveness of PTC (12). This study preoperatively scrutinized high-risk thyroid nodules confirmed as *PTC* tumors for *pTERT* mutations and inspected their relationship with clinicopathologic features at the moment of the diagnosis and co-occurrence with the *BRAF*^V600E^-mutation.

## SUBJECTS AND METHODS

### FNA specimens

We have studied 59 consecutive patients with high-risk thyroid nodules after US evaluation, followed up at the Thyroid Outpatient Clinic of the Division of Endocrinology of the Ribeirao Preto Medical School of University of São Paulo, Brazil, who needed another FNA examination and were chosen in our hospital from January 2010 to October 2012. Inclusion criteria were: (1) TIRADS 4-6 at US, or (2) TIRADS 3 that meet at least one of the following criteria: the nodule grows during follow-up (more than a 50% change in volume or a 20% increase in at least two nodule dimensions with a minimal increase of 2 mm in solid nodules or in the solid portion of mixed cystic-solid nodules), patients with higher risk of malignancy like those exposed to previous radiation to the neck or family history of DTC, and (3) histologic confirmation of PTC after thyroidectomy and elective lymph node dissection. TNM classification was built according to the American Joint Committee on Cancer (AJCC) 8^th^ edition (13,14). Genomic DNA from FNA specimens preoperatively obtained was isolated, and nested PCR was performed for direct genomic DNA sequencing to identify both the C228T and C250T *pTERT* mutations as previously described (9–11).

FNA biopsy used 24-gauge needles fitted to a 10-mL syringe. Most of the material (about two thirds) from the needle was used for cytological examination, and the remaining amount was used for DNA isolation after needle washing with 1 mL of the phosphate buffer. Then the sample was stored and frozen for future DNA extraction, using 20-50 μL of DNA extraction buffer solution (50 mM Tris buffer, pH 8.3; 1 mM EDTA, pH 8.0; 5% Tween 20 and 100 μg/mL proteinase K) with 10% resin added to the samples and incubated at 56.8 °C for a minimum of 1 hour. After incubation, the tubes were heated to 100 °C for 10 minutes, followed by centrifugation to pellet the debris, and 5 μL of the supernatant was used in the PCR reaction.

### *BRAF*^V600E^ mutation analysis

PCR was performed to amplify the exon 15 of BRAF from the isolated DNA in 20 μL reaction volume containing 100 ng of genomic DNA, 7.5 pmol of each primer, 100 μm deoxynucleoside triphosphates (dNTPs), 5 μCi [α32P] dCTP, 1.5 mm MgCl2, Platinum TaqDNA polymerase high fidelity and buffer (Thermo Fisher Scientific, Waltham, MA, USA). The primer pair was designed flanking BRAF exon 15: 5‘ AAACTCTTCATAATGCTTGCTCTG3’ (sense) and 5‘GGCCAAAAATTTAATCAGTGGA 3’ (antisense). Quality confirmation of the PCR products was achieved by gel electrophoresis, and sequencing PCR was performed using the Veriti 96-Well Thermal Cycler (Thermo Fisher Scientific, Waltham, MA, USA).

### *pTERT* mutation analysis

A fragment of the *pTERT*, which contained the sites for *pTERT*^C250T^ and *pTERT*^C228T^ mutations, was amplified by nested PCR on 50-100 ng of genomic DNA from FNA specimens. The first PCR used pair primers [5’ACGAACGTGGCCAGCGGCAG3’ (sense) and 5’CTGGCGTCCCTGCACCCTGG3’ (antisense)] in a 0,4 µM, 200 µM dNTPs, 1,5 mM MgCl_2_, PCRx Enhancer System 1X (Life Technologies, Carlsbad, CA), Taq DNA polimerase recombinant (Life Technologies, Carlsbad, CA), buffer and water (UltraPure™ DNase/RNase-Free Distilled Water, Life Technologies, Carlsbad, CA). It was performed with an initial denaturation at 94 °C for 5 minutes, followed by 40 cycles of 94 °C for 30 seconds, 62 °C annealing for 30 seconds, 72 °C elongation for 45 seconds and final completion with an elongation at 72 °C for 15 min. The second PCR used a dilution (1:50) of the first PCR product. The primers used were 5’ AGTGGATTCGCGGGCACAGA 3’ (sense) and 5’ CAGCGCTGCCTGAAACTC 3’ (antisense) in a 0,5 µM, 200 mM dNTPs, PCRx Enhancer System 1X (Life Technologies, Carlsbad, CA), Taq DNA polimerase Hot Start High Fidelity (Life Technologies, Carlsbad, CA), buffer and water to 50 µL of final volume. This PCR was performed with an initial denaturation at 98 °C for 3 minutes, followed by 35 cycles of 98 °C for 20 seconds, 66 °C annealing for 30 seconds, 72 °C elongation for 30 seconds and final completion with an elongation at 72 °C for 10 minutes.

### Statistic analysis

For analysis of the relationship between tumor clinicopathologic features and presence of *pTERT/BRAF*^V600E^ mutations, Pearson's chi-square test and Fisher's exact test were used. A linear-by-linear test was used to examine the association between T stage, N stage, AJCC stage, and mutations.

## RESULTS

A total of 59 patients with confirmed PTC was enrolled, and 14 patients were excluded due PCR failure (n = 8) and 6 due to incomplete clinical data. Therefore, 45 thyroid nodules confirmed as PTC cases were included after histological re-review by two experienced pathologists. As reported in Table 1, 39 out of 45 patients (86.7%) were women, and the mean age for all the cases was 48.5 ± 14.33 years (range 16-78). Among those, 16 patients were over 55 years old (median) at the time of diagnosis. The median tumor size was 1.9 cm (range 0.6-6.8), mean 1.35 ± 1.48 cm, with 13 (29.5%) tumors smaller than 1 cm. More than half (75.6%) of PTC were unifocal; 4 cases had a focal extra thyroidal extension, and only 7 cases had lymph node metastases at diagnosis. Four distant metastases were observed. In all, 17 (38.6%) patients had locally advanced disease (AJCC stage III or IV), and the presence of capsular invasion was observed for 8 (17.8%) patients. In all, 66.7% (30/45) and 8.9% (4/45) of the cases were *BRAF*^V600E^-mutated and TERT-mutated, respectively.

**Table 1:** Clinical and pathological characteristics of 45 patients harboring thyroid nodules confirmed as papillary thyroid carcinoma and preoperatively submitted to molecular analysis of *pTERT* and *BRAF*^V600E^ mutations

Variables		n	%
Gender			
	Male	6	13.3
	Female	39	86.7
Age, mean (sd)		48.5 (14.33)	
	≤ 55	23	51.1
	> 55	22	48.9
Tumor size			
	≤ 1 cm	13	29.5
	> 1 cm	31	70.5
Multicentricity			
	Absent	34	75.6
	Present	11	24.4
Extrathyroid extension			
	Absent	41	91.1
	Present	4	8.9
Lymphnode metastases			
	Absent	38	84.4
	Present	7	15.6
AJCC stage			
	I+II	33	75.0
	III+IV	11	25.0
Capsular invasion			
	Absent	37	82.2
	Present	8	17.8
BRAF status			
	Wild Type	15	33.3
	Mutated	30	66.7
TERT status			
	Wild Type	41	91.1
	Mutated	4	8.9

### Correlation between *pTERT*/*BRAF*^V600E^ status and clinicopathological parameters

**Figure 1:**
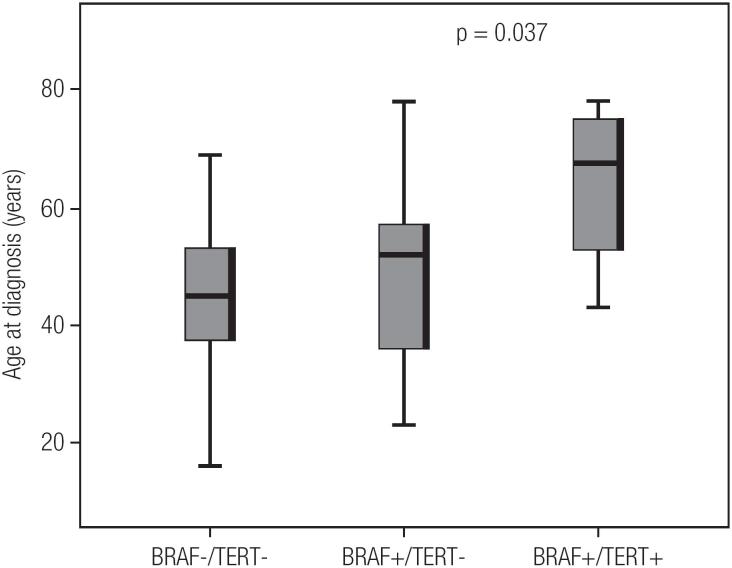
Relationship between age at PTC diagnosis and mutation status.

Four PTC tumors (two oncocytic, one classic and one trabecular) had *pTERT* mutations, and all additionally harbored the *BRAF*^V600E^ mutation. There was no association between isolated *BRAF*^V600E^ mutations and clinic-pathological parameters. However, the found *pTERT*^C228T^ mutation was independently associated with advanced age (p = 0.02) and high AJCC stage (p = 0.03) (Table 2). Interestingly, three of four patients with concomitant *BRAF*^V600E^ and *pTERT*^C228T^ mutations were classified as stage III-IV. There was significant difference in age at diagnosis between wild type for both *BRAF*^V600E^ and *pTERT*^C228T^ mutations, only *BRAF*^V600E^ positive and with concomitant *BRAF*^V600E^ and *pTERT*^C228T^ mutations patients. (p = 0.03) (Figure 1). However, in the follow-up evaluation, one patient had died, two present excellent response and another has indeterminated response to treatment at the study data snapshot.

**Table 2:** Relationship of *pTERT*/*BRAF*^V600E^ mutation and clinicopathologic aspects in 45 thyroid nodules specimens obtained by fine needle aspiration and postoperatively confirmed as papillary thyroid carcinoma

Variables		BRAF status, n (%)		P value	TERT status, n (%)		P value
		Wild type	Mutated		Wild type	Mutated	
Gender							
	Male	1 (6.7)	5 (16.7)	0.647	5 (12.2)	1 (25.0)	0.448
	Female	14 (93.3)	25 (83.3)		36 (87.8)	3 (75.5)	
Age, mean (sd)		44.0 (14.35)	50.7 (14.0)	0.139	46.9 (13.5)	64.0 (15.3)	**0.022**
Tumor size							
	≤ 1 cm	3 (21.4)	10 (33.3)	0.498	13 (32.5)	0 (0.0)	0.302
	> 1 cm	11 (78.6)	20 (66.7)		27 (67.5)	4 (100.0)	
Multicentricity							
	Absent	12 (80.0)	22 (73.3)	0.726	30 (73.2)	4 (100.0)	0.558
	Present	3 (20.0)	8 (26.7)		11 (26.8)	0 (0.0)	
Extrathyroid extension							
	Absent	14 (93.3)	27 (90.0)	1.00	38 (92.7)	3 (75.0)	0.320
	Present	1 (6.7)	3 (10.0)		3 (7.3)	1 (25.0)	
Lymphnode metastases							
	Absent	14 (93.3)	24 (80.0)	0.395	36 (87.8)	2 (50.0)	0.108
	Present	1 (6.7)	6 (20.0)		5 (12.2)	2 (50.0)	
AJCC stage							
	I+II	12 (85.7)	21 (70.0)	0.456	33 (82.5)	0 (0.0)	**0.002**
	III+IV	2 (14.3)	9 (30.0)		7 (17.5)	4 (100.0)	
Capsular invasion							
	Absent	13 (86.7)	24 (80.0)	0.699	34 (82.9)	3 (75.0)	0.557
	Present	2 (13.3)	6 (20.0)		7 (17.1)	1 (25.0)	

## CONCLUSION

We investigated the feasibility of combined *BRAF*^V600E^*/pTERT* mutations testing on routine FNA specimens and its prognostic value in US guide biopsied of high suspicious thyroid nodules. The *BRAF*^V600E^ and *pTERT* mutations were found in a frequency of 66.7% and 8.9%, respectively. Indeed, we observed that *BRAF*^V600E^*/pTERT* mutation-positive thyroid nodules were detected only in cancers that behaved aggressively, representing 4/11 (36%) of advanced stage PTCs and harboring threatening clinic-pathological features such as lymph node metastases, extra-thyroidal invasion and distant metastases (Table 3).

**Table 3:** Correlation of *pTERT*/*BRAF*^V600E^ mutations and clinicopathologic aspects in 45 thyroid nodules specimens obtained by fine needle aspiration and postoperatively confirmed as papillary thyroid carcinoma

Variables		BRAF/TERT status, n (%)		
		BRAF-/TERT-	BRAF+/TERT-	BRAF+/TERT+
Gender				
	Male	14 (93.3)	22 (84.6)	3 (75.0)
	Female	1 (6.7)	4 (15.4)	1 (25.0)
		P value^a^ = 0.407	P value^b^ = 0.636	P value^c^ = 0.386
Age, mean (sd)		44.0 (14.35)	48.7 (12.9)	64.0 (15.3)
		P value^a^ = **0.042**	P value^b^ = 0.881	P value^c^ = **0.037**
Tumor size				
	≤ 1 cm	3 (21.4)	10 (38.5)	0 (0.0)
	> 1 cm	11 (78.6)	16 (61.5)	4 (100.0)
		P value^a^ = 0.284	P value^b^ = 0.316	P value^c^ = 1.00
Multicentricity				
	Absent	12 (80.0)	18 (69.2)	4 (100.0)
	Present	3 (20.0)	8 (30.8)	0 (0.0)
		P value^a^ = 0.592	P value^b^ = 0.716	P value^c^ = 1.00
Extrathyroid extension				
	Absent	14 (93.3)	24 (92.3)	3 (75.0)
	Present	1 (6.7)	2 (7.7)	1 (25.0)
		P value^a^ = 0.509	P value^b^ = 1.00	P value^c^ = 0.386
Lymphnode metastases				
	Absent	14 (93.3)	22 (84.6)	2 (50.0)
	Present	1 (6.7)	4 (15.4)	2 (50.0)
		P value^a^ = 0.139	P value^b^ = 0.636	P value^c^ = 0.097
AJCC stage				
	I+II	12 (85.7)	21 (80.8)	0 (0.0)
	III+IV	2 (14.3)	5 (19.2)	4 (100.0)
		**P value** a **= 0.004**	P value^b^ = 1.00	P value^c^ = **0.005**
Capsular invasion				
	Absent	13 (86.7)	21 (80.8)	3 (75.0)
	Present	2 (13.3)	5 (19.2)	1 (25.0)
		P value^a^ = 0.861	P value^b^ = 1.00	P value^c^ = 0.530

a Global test.

b BRAF-/TERT- vs BRAF+/TERT- comparasion.

c BRAF-/TERT- vs BRAF+/TERT+ comparasion.

We did not evaluate the degree of concordance between matched FNA and formalin-fixed, paraffin-embedded samples. The mutation analysis sensitivity can be compromised by using lavage fluid once the amount and composition of the cellular content is unknown, potentially leading to discordance between matched FNA and formalin-fixed, paraffin-embedded. As a result, we were not able to measure the mutation false-negative and false-positive rates on FNA preparations. Indeed, strategies such as real-time Light Cycler PCR and fluorescence melting curve analysis might be superior.

In conclusion, preoperative determination of *BRAF*^V600E^ and *pTERT* mutations status can be easily performed on cytologic preparation using lavage fluids collected from needle rinsing. The presence of the *BRAF*^V600E^*/pTERT* mutations could be a marker of poor prognosis in elderly population, although the absence of the mutation may not yet be considered an index of good prognosis to individualize treatment decisions and follow-up protocol. Preoperative knowledge of the *BRAF*^V600E^*/pTERT* mutations status would help determine the extent of surgery for thyroid nodules. Disease free or overall survival is still unclear.
